# Dual-center validation of using magnetic resonance imaging radiomics to predict stereotactic radiosurgery outcomes

**DOI:** 10.1093/noajnl/vdad064

**Published:** 2023-05-27

**Authors:** David A DeVries, Terence Tang, Ghada Alqaidy, Ali Albweady, Andrew Leung, Joanna Laba, Frank Lagerwaard, Jaap Zindler, George Hajdok, Aaron D Ward

**Affiliations:** Department of Medical Biophysics, Western University, London, ON, Canada; Gerald C. Baines Centre, London Health Sciences Centre, London, ON, Canada; Department of Radiation Oncology, London Regional Cancer Program, London, ON, Canada; Radiodiagnostic and Medical Imaging Department, King Fahad Armed Forces Hospital, Jeddah, Saudi Arabia; Department of Radiology, Unaizah College of Medicine and Medical Sciences, Qassim University, Unaizah, Saudi Arabia; Department of Medical Imaging, Western University, London, ON, Canada; Department of Radiation Oncology, London Regional Cancer Program, London, ON, Canada; Department of Oncology, Western University, London, ON, Canada; Department of Radiation Oncology, Amsterdam University Medical Centre, Amsterdam, The Netherlands; Department of Radiation Oncology, Haaglanden Medical Centre, Den Haag, The Netherlands; Holland Proton Therapy Centre, Delft, The Netherlands; Department of Medical Biophysics, Western University, London, ON, Canada; Department of Medical Biophysics, Western University, London, ON, Canada; Gerald C. Baines Centre, London Health Sciences Centre, London, ON, Canada; Department of Oncology, Western University, London, ON, Canada

**Keywords:** brain metastasis, machine learning, magnetic resonance imaging, radiomics, stereotactic radiosurgery

## Abstract

**Background:**

MRI radiomic features and machine learning have been used to predict brain metastasis (BM) stereotactic radiosurgery (SRS) outcomes. Previous studies used only single-center datasets, representing a significant barrier to clinical translation and further research. This study, therefore, presents the first dual-center validation of these techniques.

**Methods:**

SRS datasets were acquired from 2 centers (*n* = 123 BMs and *n* = 117 BMs). Each dataset contained 8 clinical features, 107 pretreatment T1w contrast-enhanced MRI radiomic features, and post-SRS BM progression endpoints determined from follow-up MRI. Random decision forest models were used with clinical and/or radiomic features to predict progression. 250 bootstrap repetitions were used for single-center experiments.

**Results:**

Training a model with one center’s dataset and testing it with the other center’s dataset required using a set of features important for outcome prediction at both centers, and achieved area under the receiver operating characteristic curve (AUC) values up to 0.70. A model training methodology developed using the first center’s dataset was locked and externally validated with the second center’s dataset, achieving a bootstrap-corrected AUC of 0.80. Lastly, models trained on pooled data from both centers offered balanced accuracy across centers with an overall bootstrap-corrected AUC of 0.78.

**Conclusions:**

Using the presented validated methodology, radiomic models trained at a single center can be used externally, though they must utilize features important across all centers. These models’ accuracies are inferior to those of models trained using each individual center’s data. Pooling data across centers shows accurate and balanced performance, though further validation is required.

**Key Points**

- Models were trained at 2 centers using identical methods, demonstrating external validation.
- Feature harmonization and consolidation are required for model transfer across centers.
- Training with pooled data offers balanced performance for either center.

**Importance of the Study**

Using MRI radiomics and machine learning to predict brain metastasis (BM) SRS outcomes would be of clinical benefit, but validation of the proposed models and methodologies beyond single-center studies is critical to motivate research and clinical translation. Our study presents the first such dual-center external validation. We first show that feature harmonization and consolidation techniques are required to increase accuracy when training a model at one center for use at another. We also show that accuracy was maintained when taking a locked model training methodology developed at one center and using it to train a model at another center. This provides a validated methodology for other centers to train their own predictive models. Lastly, we showed that the use of pooled data from both centers offers balanced predictive performance for either center, demonstrating a possible method to obtain a more generalizable model.

Approximately 10%–20% of cancer patients develop brain metastases (BM), the spread of cancer to the brain.^1^ With cancer treatment improvements, both BM incidence and BM patient life expectancy have increased. However, the overall prognosis remains poor, with a median overall survival between 8 and 16 months.^2^ As a result, BM patients must be treated quickly and effectively, while minimizing short and long-term toxicities.

Stereotactic radiosurgery (SRS) represents an effective BM treatment, in which ablative radiation doses are delivered conformally in 1 to 3 fractions to limit normal tissue toxicity.^3^ Stereotactic radiation therapy (SRT), a variant of SRS, delivers hypofractionated radiation over 5 fractions to larger BMs or surgical cavities. SRS has important advantages over alternative BM treatment options. Compared to whole-brain radiation therapy, where radiation is delivered to the entire brain, SRS limits normal brain tissue irradiation to reduce the risk of long-term cognitive side effects that negatively impact patients’ quality of life.^4^ Compared to surgery, SRS is noninvasive and so avoids standard surgical risks.

Despite the ablative nature of SRS, it is associated with treatment failure rates up to 30%, in which the targeted BMs progress post-SRS.^4^ Higher prescription doses could decrease failure rates, but would also increase the risk of toxicities.^5–7^ Knowing whether SRS is likely to fail at a given dose prescription may help to drive decisions to treat with higher prescription doses, balancing this against the risk of toxicities. Therefore, having predictive models of BM SRS response could aid in treatment selection and optimizing SRS dose prescription.

Previous studies have demonstrated an association between qualitative BM appearance in T1-weighted contrast-enhanced magnetic resonance imaging (T1w-CE MRI) and SRS outcomes.^8,9^ Specifically, it has been hypothesized that hypointense areas following contrast injection may indicate areas of hypoxia with reduced radiosensitivity.

Recently, quantitative machine learning (ML) techniques have also been investigated to predict SRS outcomes. These techniques rely on quantitative radiomic features extracted from medical images and ML models to make treatment predictions.^10,11^ These studies have produced accurate predictive models incorporating a variety of MRI sequences and clinical data points.^12–18^ Similar studies have also predicted SRT or post-resection SRS outcomes.^19–24^ However, all these studies have used single-center datasets, leaving the generalizability of these models and techniques an open question.

To motivate clinical translation of ML techniques, multi-center external validation must be performed to demonstrate generalizability.^25^ It is critical to first validate that a model trained at one center can produce accurate predictions at another center, as this represents the most straightforward route towards clinical deployment. Alternatively, each center could train its own model, but this requires external validation of the model training methodology to ensure it reliably creates accurate models at independent centers. We therefore provide the first external validation of ML-based SRS outcome prediction by answering the following research questions:

1. Can a model trained with one center’s dataset make accurate predictions on another center’s dataset without retraining?
2. Can the methodology used to produce a model from one center’s dataset be successfully used to produce a similarly performing model from another center’s data?
3. Does a model trained on pooled data from 2 centers offer equivalent accuracy for each center?
4. When 2 models are trained using an identical methodology, but at different centers, are the same clinical and radiomic features found to be important?
5. Can the accuracy of models described in research question #1 be improved by either harmonizing feature values across centers, or by utilizing a consolidated set of features that are mutually important across both centers?

## Methods

### Center A Study Sample

To answer our research questions, we acquired datasets from study samples at 2 centers. The first study sample was provided by the Amsterdam University Medical Center (AUMC) in the Netherlands (defined as “Center A”) and was the identical 99-patient sample used in our previous ML study.^17^ The AUMC Medical Ethical Review Committee approved this retrospective data collection, with participant consent waived due to the study being retrospective and on deceased patients.

Center A’s dataset consisted of imaging and clinical data points collected per BM. The retrospective study sample inherently reflects Center A’s clinical practice when the patients were treated (2003–2011). Our study’s inclusion and exclusion criteria restricted patients to have received first-line SRS and at least one follow-up MRI. The AUMC only used linear accelerator-based (linac-based) SRS, with prescriptions ranging from 15 to 24 Gy in 1 or 3 fractions (see Table 1). *n* = 123 BMs across the 99 patients were individually analyzed, with demographics provided in Table 1. pretreatment T1w-CE MRI was collected per BM, along with the gross tumor volume (GTV) contour used for treatment planning as defined by an experienced clinician using the enhancing region’s border. Five MR scanner models were represented in the dataset, with 94% of BMs imaged across 3 models (see Supplementary Table S1).

**Table 1.** Univariate Analysis of Clinical and Radiomic Features for Centers A and B to Separate BMs That Progressed Post-SRS From Those That Did Not, and Separation of Center A and Center B BMs

Features		Center A		Center B		Centers A & B Pooled	
Categorical clinical features		# BMs (% +)	+ vs. − *P*-value	# BMs (% +)	+ vs. − *P*-value	+ vs. − *P*-value	A vs. B *P*-value
Sex							
*Female*		66 (21.2%)	.83	71 (16.9%)	.95	.79	.27
*Male*		57 (22.8%)		46 (17.4%)			
Primary cancer site							
*Lung*		70 (12.9%)	.001	89 (16.9%)	.21	.001	.005
*Breast*		14 (35.7%)		10 (20.0%)			
*Skin*		9 (66.7%)		8 (37.5%)			
*Other*		30 (23.3%)		10 (0.0%)			
Primary cancer histology							
*Adenocarcinoma*		65 (20.0%)	.001	82 (17.1%)	.55	.002	.0006
*NSCLC Other*		36 (11.1%)		9 (11.1%)			
*Melanoma*		9 (66.7%)		8 (37.5%)			
*Squamous*		8 (50.0%)		8 (12.5%)			
*Other*		5 (0.0%)		10 (10.0%)			
Systemic therapy status							
*Yes*		113 (22.1%)	.88	57 (10.5%)	.07	.41	<.0001
*No*		10 (20.0%)		60 (23.3%)			
Prescription fractions							
*1*		113 (18.6%)	.002	58 (15.5%)	.65	.21	<.0001
*3*		10 (60.0%)		59 (18.6%)			
**Continuous** **clinical features**		**Median (range)**	**+ vs. −** ***P*-value**	**Median (range)**	**+ vs. −** ***P*-value**	**+ vs. −** ***P*-value**	**A vs. B** ***P*-value**
Age *(years)*		58.5 (38.4-86.0)	.92	67.8 (43.6-82.2)	.72	.66	<.0001
BM volume *(mm*^*3*^)		3500 (100-41200)	.0003	329 (12-5564)	.003	<.0001	<.0001
Prescription dose (*Gy)*		21 (15-24)	.63	21 (18-27)	.56	.95	<.0001
**Radiomic features (# of features)**		**# Features + vs. − *P*-value <.05 (% of features)**		**# Features + vs. − *P*-value <.05 (% of features)**		**# Features *P*-value <.05 (% of features)**	
						**+ vs. −**	**A vs. B**
First-Order	(18)	2 (11.1%)		9 (50.0%)		4 (22.2%)	10 (55.6%)
Shape & Size	(14)	11 (78.6%)		11 (78.6%)		11 (78.6%)	14 (100.0%)
GLCM	(24)	10 (41.7%)		11 (45.8%)		10 (41.7%)	14 (58.3%)
GLRLM	(16)	4 (25.0%)		10 (62.5%)		14 (87.5%)	14 (87.5%)
GLDM	(14)	6 (37.5%)		9 (64.3%)		11 (78.6%)	13 (92.9%)
GLSZM	(16)	7 (43.8%)		10 (62.5%)		13 (81.3%)	14 (87.5%)
NGTDM	(5)	4 (80.0%)		2 (40.0%)		4 (80.0%)	4 (80.0%)
*Total*	*(107)*	*44 (41.1%)*		*62 (57.9%)*		*67 (62.6%)*	*83 (78.6%)*

*P*-values were calculated using the Chi-squared test for the categorical clinical features, while the Wilcoxon rank sum test was used for the continuous clinical features and radiomic features. “+ vs. **−***P*-value” represents testing between BMs that progressed post-SRS (“+”) and those that did not (“**−**“). “A vs. B *P*-value” represents testing between all BMs from Center A against all BMs from Center B. Given the large number of radiomic features, the number of features below α = 0.05 is given for each class of radiomic feature, along with percentage of features from each class this number of features represents. A full table of all features’ *P*-values are provided in Supplementary Material.

NSCLC, non-small cell lung cancer; GLCM, Gray-Level Co-occurrence Matrix; GLRLM, Gray-Level Run Length Matrix; GLDM, Gray-Level Dependence Matrix; GLSZM, Gray-Level Size Zone Matrix; NGTDM, Neighbouring Gray Tone Difference Matrix.

We defined BM post-SRS progression radiographically using follow-up MRI approximately every 3 months. The AUMC’s data collection occurred before the introduction of the Response Assessment in Neuro-Oncology Brain Metastases (RANO-BM) protocol,^26^ and so BM size was defined by measuring the maximum diameter in 3 perpendicular directions (superior–inferior, mediolateral, posterior–anterior). The product of the maximum diameters provided an approximation of BM volume, with an increase of ≥ 25% indicating progression. Post-SRS progression was therefore defined as a binary label, with a positive (“+”) representing a BM that progressed post-SRS, and negative (“−“) representing no progression. BMs can display pseudo-progression, radiographic growth not linked to cancerous progression.^6^ Pseudo-progression was controlled for based on expert clinician judgment using MRI and patient medical records. In total, 22.0% of Center A’s BMs were scored as true progression.

### Center B Study Sample

We collected a second, external dataset retrospectively at the London Regional Cancer Program in Canada (“Center B”). First-line SRS and at least one follow-up MRI were also required. Linac-based SRS was also solely used in 1 or 3 fractions with doses of 18–27 Gy (see Table 1). *n* = 117 BMs across 62 patients treated in 2016–2020 were included. GTV contours and T1w-CE MRI were also collected, across which 5 MR scanner models were represented, with 55% and 30% of BMs imaged across 2 models (Supplementary Table S1). The Western University Research Ethics Board approved this study and waived patient consent, and data collection used the REDCap platform.^27,28^

To maintain consistency, we used Center A’s definition of progression for Center B. However, Center B’s dataset contained smaller BMs than Center A’s (Center A contained no BMs of volume <100 mm^3^). A percentage-based progression metric was inappropriate for Center B’s BMs <100 mm^3^, as an insignificant change in absolute BM size would still represent a ≥25% change. This limitation in percentage-based scoring for small BMs was also noted in the RANO-BM protocol for unidimensional measurements, with a 3 mm absolute change in the longest BM diameter recommended by RANO-BM instead.^26^ We adapted this technique for 3-dimensional measurements, with a (3 mm)^3^ = 27 mm^3^ change in size required to constitute progression for BMs in which a 25% change in volume was < 27 mm^3^. We controlled for pseudo-progression using the trajectory of BM size, salvage therapies, and pathology reports (Supplementary Table S2). In total, 17.1% of Center B’s BMs progressed post-SRS. Five BMs were particularly challenging to assess for progression due to immunotherapy or targeted therapies (Supplementary Table S2). These BMs could have possibly confounded model accuracy, and so we repeated Center B’s methodology external validation experiments described below without these BMs. The results changed negligibly, and so we included these BMs.

### Clinical Features

We also collected pretreatment clinical features at both centers. As the Center A and B datasets were retrospective, each center’s clinical features did not directly overlap, and so we used a common set of 8 clinical features (listed in Table 1). The presence of primary cancer site and histology feature categories at one center and not the other was accounted for using an “Other” category. We investigated separation of BMs from each center and of positive and negative BMs using per-clinical-feature univariate statistical tests (Table 1).

### Radiomic Features

Before radiomic feature extraction, we preprocessed the pretreatment T1w-CE MRI to account for variability in voxel size and intensity scaling. Specifically, we computed the mean and standard deviation of the voxels within the brain and not the BMs to apply Z-score normalization to the MR scan at 3 standard deviations.^29^ The voxels were then linearly interpolated to a size of 0.5 × 0.5 × 0.5 mm^3^. After preprocessing, 107 radiomic features were extracted for each BM’s GTV region-of-interest (feature list in Supplementary Table S3) using PyRadiomics library v3.0.1 in Python v3.6.13^30^ with 64 intensity value bins. Univariate statistical tests were also performed on the radiomic features (Table 1).

### ML Methodology

For each ML experiment, we used Matlab 2019b v9.7.0.1190202 (The Mathworks Inc., Natick, USA) to train random decision forest (RDF) models, with hyperparameter optimization performed before training. We explored 3 feature combinations: clinical features, radiomic features, and all features (clinical and radiomic). If radiomic features were included, an inter-feature correlation filter removed redundant features with a correlation coefficient >0.8. Some experiments had a single training dataset from one center and a single testing dataset from the other center, while some experiments required using one dataset that was bootstrap resampled into training and testing datasets over 250 repetitions. We developed this methodology in our previous study based only on Center A’s dataset,^17^ with further detail provided in Supplementary Figure S1 and Table S4.

From each experiment’s testing dataset, we calculated receiver operating characteristic (ROC) curves and the associated error metrics of area under the ROC curve (AUC), misclassification rate (MCR), false negative rate (FNR), and false positive rate (FPR). For non-bootstrap experiments, ROC curves were constructed for both the testing dataset’s samples and the training dataset’s RDF out-of-bag samples. To avoid positive bias from using the testing dataset, we used the training dataset’s ROC to choose the upper-left operating point, minimizing the sum of squares of FNR and FPR. We transferred the operating point to the testing dataset’s ROC to allow for the calculation of MCR, FNR, and FPR, with the AUC calculated from the testing ROC itself. For bootstrap experiments, the process was similar, except that the 250 repetitions allowed for the calculation of average ROC curves and error metrics with associated 95% confidence intervals (CIs). The average AUC from bootstrap resampling underestimates the true expected AUC, and so we separately reported the common AUC_0.632+_ correction.^31^

### Model External Validation

**Figure 1.**
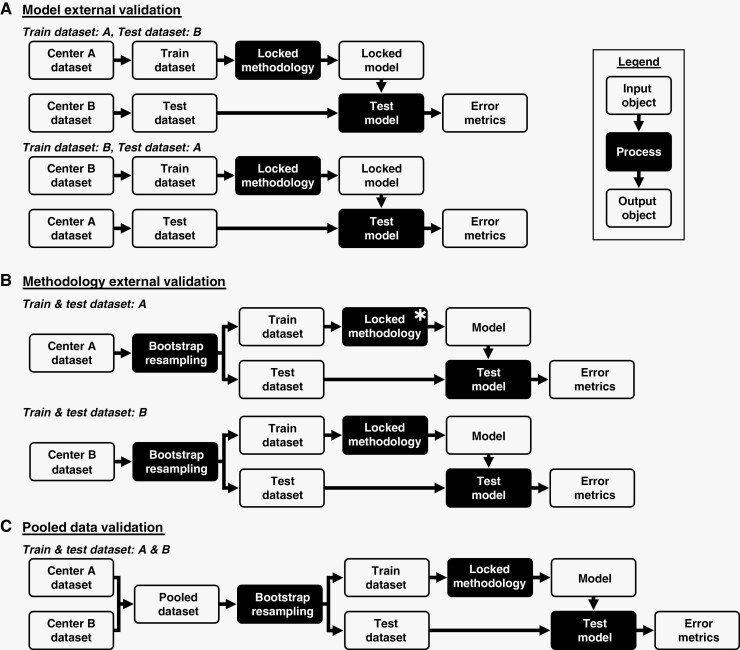
Visual representation of the 3 types of validation performed. (A) depicts model external validation, in which a model is trained using Center A’s dataset, locked, and then tested using Center B’s dataset. The inverse in which Center A’s and B’s datasets are exchanged was also performed, as shown. (B) shows methodology external validation, in which a locked methodology is used to train and test models using Center B’s dataset via bootstrap resampling, as shown under “Train & Test Dataset: B.” “Train & Test Dataset: A” shows a similar technique employed to develop the original model training methodology using only Center A’s dataset that was then locked (indicated by the “*”) and then used and externally validated in the other experiments. “Train & Test Dataset: A” was also used in this study to revalidate the locked methodology with Center A’s dataset. Pooled data validation in (C) is identical to (B), except that Center A and B’s datasets are pooled before bootstrap resampling.

To address research questions 1–3, we conducted 3 types of validation. The first type, “model external validation,” answered research question 1 by training a model with one center’s dataset (either A or B), and then testing it with the other center’s dataset. As shown in Figure 1A, this validation type is the most rigorous, with a locked model being tested at another center.

### Methodology External Validation

To address research question 2, we performed “methodology external validation” by taking the locked methodology to train a model developed using Center A’s dataset and applying it to Center B’s dataset via bootstrap resampling to validate training a model per-center using a methodology developed externally. The methodology was “locked” in the sense that all design decisions for how models are trained and have their hyperparameters optimized were held constant. Figure 1A,B compares this “methodology external validation” to the previous “model external validation.”

### Pooled Data Validation

Lastly, to answer research question 3, we combined both centers’ datasets to perform “pooled data validation.” Figure 1C shows how bootstrapped resampling allows for data from Center A and B to be represented in distinct training and testing datasets. We calculated overall and per-center error metrics to evaluate if model accuracy was consistent for both centers’ testing samples.

### Feature Importance Analysis

Based on each center’s methodology external validation results, we performed feature importance analysis to answer research question 4. RDFs inherently supply feature importance scores, which were normalized between 0 and 1 for each bootstrap iteration. Radiomic features removed by the inter-feature correlation filter were unused by the RDFs, and so were assigned a score of 0. The scores were then averaged across bootstrap repetitions and re-normalized to be between 0 and 1.

### Feature Harmonization and Consolidation

To explore the feature harmonization proposed in research question 5, we used “combating batch effects when combining batches” (ComBat) harmonization.^32^ MRI acquisition parameters and scanner model are known to systematically affect radiomic features.^29,33^ While our MRI preprocessing can mitigate these effects, ComBat harmonization provides further corrections after feature extraction. ComBat finds multiplicative and additive terms that represent the effect of data being collected at a given center, and then their inverse is applied to minimize inter-center differences.^32,34^ We therefore repeated the model external validation (Figure 1A) using only radiomic features after applying ComBat.

We also hypothesized that radiomic feature variation could be mitigated through “consolidation,” by only including features that are important at both centers. The idea behind consolidation is that there may be features that are uniquely important at one center, but also other features that are important across all centers. For our 2 centers, the previous feature importance analysis provided lists of radiomic features ranked by importance. From these lists, the top 10 commonly important features were consolidated using a ranking metric of the lowest importance score a feature received across the 2 centers. We then conducted model external validation experiments using only the top *n* consolidated radiomic features with ComBat harmonization also applied, with *n* ranging from 1 to 10.

## Results

### Model External Validation

**Figure 2.**
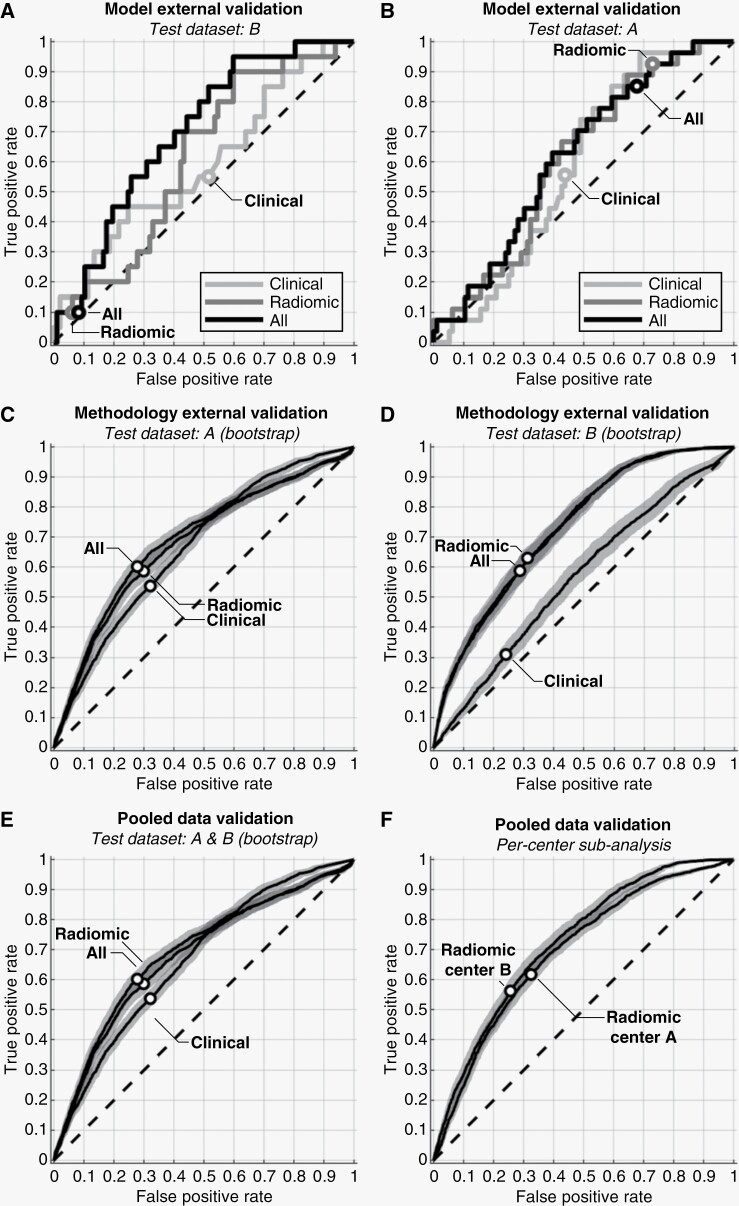
ROC curves and operating points for validation experiments. ROC curves for model external validation are provided when using Center B’s dataset (A) or Center A’s dataset (B) for testing. ROC curves when using only clinical features (“Clinical”), only radiomic features (“Radiomics”), or both clinical and radiomic features (“All”) are labeled, with each curve’s operating point marked by a “○.” No CIs are provided in these cases, as a single testing dataset was used. (C) and (D) provide methodology external validation ROC curves for Center A and B, respectively (shaded bands represent the 95% CIs). Pooled data validation ROC curves are shown in (E), with (F) showing the per-center sub-analysis when using only radiomic features. For all ROCs, associated error metrics are quantified in Table 2, with the right-most “ROC Figure” column of Table 2 allowing for cross-referencing.

The model trained on Center A’s clinical features and then tested on Center B produced an AUC of 0.59 and MCR of 50.4%. The ROC curve from this experiment is provided in Figure 2A, with the error metrics provided in Table 2 (first row of section A). The inverse scenario of training a model on Center B and testing on Center A also yielded low accuracy (ROC curve in Figure 2B; error metrics in Table 2, fourth row of section A). No CIs are associated with these model external validation results, as only a single model was tested per experiment.

**Table 2.** Error Metrics Values Across the Validation Experiments Performed

	Datasets		Features	AUC	AUC_0.632+_	MCR %	FNR %	FPR %	ROC Figure
	Train	Test							
**A: Model external validation**									
□	A	B	Clinical	0.59	-	50.4	45.0	51.5	Figure 2a
			Radiomic	0.61	-	20.5	90.0	6.2	
			All	0.70	-	22.2	90.0	8.2	
■	B	A	Clinical	0.59	-	43.9	44.4	43.8	Figure 2b
			Radiomic	0.61	-	58.5	7.4	72.9	
			All	0.62	-	56.1	14.8	67.7	
**B: Methodology external validation**									
	A (Bootstrap)		Clinical	0.67 (0.01)	0.73	35.3 (1.4)	46.3 (1.8)	32.1 (1.3)	Figure 2c
			Radiomic	0.68 (0.01)	0.74	32.4 (1.5)	41.4 (2.1)	29.9 (1.4)	
			All	0.69 (0.01)	0.76	30.5 (1.5)	39.9 (2.0)	27.8 (1.3)	
	B (Bootstrap)		Clinical	0.56 (0.01)	0.64	31.8 (1.5)	68.9 (2.4)	24.1 (1.3)	Figure 2d
			Radiomic	0.73 (0.01)	0.80	32.3 (1.7)	37.0 (2.9)	31.3 (1.4)	
			All	0.73 (0.01)	0.80	30.9 (1.6)	41.3 (3.0)	28.8 (1.3)	
**C: Pooled data validation**									
	A & B (Bootstrap)		Clinical	0.61 (0.01)	0.67	36.1 (1.1)	53.3 (1.5)	31.9 (1.0)	Figure 2e
			Radiomic	0.71 (0.01)	0.78	32.4 (1.1)	38.7 (1.7)	30.9 (1.0)	
			All	0.71 (0.01)	0.78	31.3 (1.2)	39.7 (1.8)	29.3 (1.1)	
	A & B (A Sub-Analysis)		Clinical	0.62 (0.01)	-	37.2 (1.5)	50.3 (1.9)	34.0 (1.3)	Supplementary Figure S2a
			Radiomic	0.69 (0.01)	-	33.6 (1.4)	38.3 (2.0)	32.5 (1.3)	Figure 2f
			All	0.70 (0.01)	-	32.7 (1.4)	39.1 (2.0)	31.2 (1.3)	Supplementary Figure S2b
	A & B (B Sub-Analysis)		Clinical	0.59 (0.02)	-	33.8 (1.5)	61.3 (2.4)	27.1 (1.3)	Supplementary Figure S2a
			Radiomic	0.73 (0.01)	-	29.2 (1.5)	43.8 (2.5)	25.6 (1.2)	Figure 2f
			All	0.72 (0.01)	-	30.6 (1.5)	41.0 (2.5)	28.0 (1.3)	Supplementary Figure S2b
**D: Feature harmonization**									
□	A	B	ComBat Radiomic	0.63	-	37.6	50.0	35.1	Supplementary Figure S2c
■	B	A		0.61	-	43.9	44.4	43.8	Supplementary Figure S2d
**E: Feature harmonization and consolidation**									
	A	B	*n* = 1 ComBat Radiomic	0.66	-	42.7	35.0	44.3	-
	B	A		0.63	-	49.6	18.5	58.3	-
	A	B	*n* = 2 ComBat Radiomic	0.61	-	48.7	30.0	52.6	-
	B	A		0.59	-	36.6	63.0	29.2	-
	A	B	*n* = 3 ComBat Radiomic	0.70	-	33.3	40.0	32.0	Supplementary Figure S2e
	B	A		0.65	-	30.1	63.0	20.8	
□	A	B	*n* = 4 ComBat Radiomic	0.67	-	45.3	40.0	46.4	Figure 4b
■	B	A		0.70	-	26.0	33.3	24.0	
	A	B	*n* = 5 ComBat Radiomic	0.68	-	45.3	35.0	47.4	Supplementary Figure S2f
	B	A		0.65	-	30.1	51.9	24.0	
	A	B	*n* = 6 ComBat Radiomic	0.66	-	31.6	35.0	30.9	-
	B	A		0.65	-	36.6	37.0	36.5	-
	A	B	*n* = 7 ComBat Radiomic	0.46	-	66.7	35.0	73.2	-
	B	A		0.61	-	46.3	37.0	49.0	-
	A	B	*n* = 8 ComBat Radiomic	0.60	-	35.0	50.0	32.0	-
	B	A		0.62	-	37.4	44.4	35.4	-
	A	B	*n* = 9 ComBat Radiomic	0.62	-	35.0	50.0	32.0	-
	B	A		0.58	-	47.2	40.7	49.0	-
	A	B	*n* = 10 ComBat Radiomic	0.62	-	42.7	40.0	43.3	-
	B	A		0.58	-	39.0	63.0	32.3	-

The rows of the table are divided into 5 sections (A–E), corresponding to the 3 types of validation performed, along with 2 sections regarding the feature harmonization and consolidation results. The “Features” column indicates which features were available for use by the model. Comparisons between sections should therefore be made between rows with the same features indicated, with “Radiomic” features being used across all sections. For section E, the “Features” column value refers to using the top 1 to n consolidated radiomic features with ComBat harmonization also applied. Rows marked with “□” represent model external validation experiments for comparison with Center B as the test dataset using only radiomic features with no or different feature harmonization and consolidation techniques applied. Rows marked with “■” are represent a similar comparison, but with Center A as the test dataset. The “ROC Figure” column provides a means to cross-reference error metric values with the ROC curves from which they were derived (see Supplementary Figure S2). Where bootstrapped experiments were performed, 95% CI values are supplied in parentheses, along with AUC_0.632+_ Values

Figure 2A,B and Table 2 (section A) show the ROC curves and error metrics from the model external validation using only radiomic features and using all features. AUC values were near those from using clinical features only, except when using all features from Center A to train a model, which had the highest AUC of 0.70. The ROC operating points determined from the training datasets were inappropriate, leading to highly imbalanced FNRs and FPRs.

### Methodology External Validation

We revalidated the model training methodology developed with Center A’s dataset in our previous study^17^ due to the adjusted clinical feature categories and reduced number of clinical features from 12 to 8 in Center A’s dataset required to match Center B. We found negligible differences compared to the previous study (Figure 2C; Table 2, section B), validating the methodology and adjusted clinical features, and providing a comparison baseline for Center B.

Methodology external validation using Center B’s dataset demonstrated slightly elevated accuracy compared to Center A, except when using clinical features alone where a drop of 0.09 in AUC_0.632+_ was seen (Figure 2D; Table 2, section B). Using only radiomic features or all features provided equivalent performance, with equal AUC_0.632+_ values of 0.80 and overlapping MCR, FNR, and FPR 95% CIs.

### Pooled Data Validation

The pooled data validation produced accuracy values between those of Center A’s and Center B’s methodology external validation results (Figure 2E; Table 2, section C, first 3 rows). Using only radiomic features and all features offered equivalent accuracy, matching Center B’s methodology external validation results (compare against Figure 2D). AUC_0.632+_ values for all feature combinations fell approximately halfway between Center A’s and Center B’s external methodology validation AUC_0.632+_ values.

Per-center analysis revealed that a model trained on both centers’ datasets provided balanced performance for each center (Table 2, section C for all error metrics; Figure 2F for radiomic features only ROCs). The AUC differences between the per-center error metrics were small (0.02–0.04) with non-overlapping CIs.

### Feature Importance Analysis

**Figure 3.**
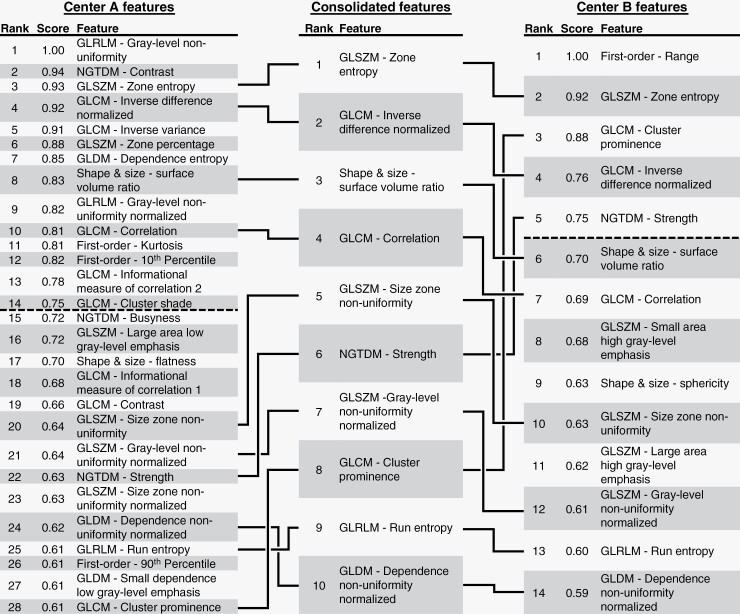
Per-center radiomic feature importance analysis and associated feature consolidation. The left and right-most tables show the most important radiomic features for Centers A and B, respectively, as computed using the methodology external validation results. The central table shows the ranking of the top 10 consolidated features from Centers A and B, with a feature’s ranking determined by taking the minimum of the importance score it received at each center. Only the features down to the least important feature required to create the top 10 consolidated features are shown, with an exhaustive feature list provided in Supplementary Data. The dashed lines represent the 0.75 cutoff for “highly important” features.

Methodology external validation radiomic feature importance analysis found commonalities and differences between Center A and B. Features with an importance score of 0.75 or greater were deemed “highly important,” with 14 such features revealed for Center A and 5 for Center B, as shown in Figure 3’s left and right-most tables (features above dashed line). At both centers, primarily higher-order texture features were important, and in particular, gray-level co-occurrence matrix (GLCM) features (33.3% of Center A’s important features; 40.0% of Center B’s). First-order statistical features were in the minority at both centers, and only Center A used a highly important shape and size-based feature. Only 2 features, gray-level size zone matrix zone entropy and GLCM inverse difference normalized, were highly important at both centers. Importance scores for all radiomic features are provided in the Supplementary data.

### Feature Harmonization and Consolidation

ComBat feature harmonization was found to have a minimal impact on model performance. ComBat harmonization on the radiomic features caused model external validation AUC values to remain unchanged or rise slightly by 0.02 (see indicated comparison between values in Table 2, sections A, D). The chosen ROC operating points chosen were more appropriate, however (Table 2, section D). Feature harmonization was pursued only with radiomic features, as the univariate predictive value of clinical features differed greatly between the 2 centers (see Table 1), and clinical features added no benefit to Center B’s methodology external validation.

A consolidated set of the top 10 mutually important features was then established, as shown in the middle table of Figure 3. These consolidated features consisted almost entirely of higher-order texture features, with one shape and size feature included. GLCM and gray-level size zone matrix features made up the majority of the set, including 4 of the top 5 features. To construct the feature set, features with importance scores down to near 0.60 were required, as shown through the linkages between tables in Figure 3.

**Figure 4.**
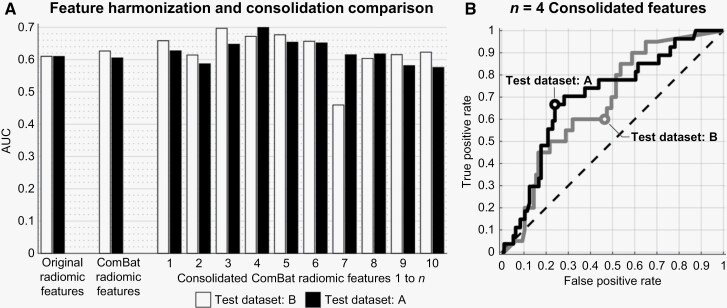
Comparison of applying radiomic feature harmonization and consolidation against baseline, and ROC curves when using the optimal top *n* consolidated features. (A) compares the baseline model external validation AUC results against applying only ComBat harmonization and against using 1 to *n* of the top 10 consolidated features with ComBat harmonization also applied. (B) shows the ROC curves for the optimal number of consolidated features, *n* = 4. Full error metric quantification for (A) and (B) is provided in Table 2 within sections A, D, and E.

When using the top *n* = 4 of the consolidated features, the highest average AUC across testing on Center A or B was achieved (Figure 4A; further error metrics in Table 2, section E). The AUC values from each center were within 0.03, while the associated MCR, FPR, and FNR were quite different. Figure 4B demonstrates this is due to ROC operating point selection, with the ROC curves demonstrating multiple points of equivalent FPR and FNR across centers.

## Discussion

Our study demonstrates the generalizability across 2 independent centers of post-SRS progression predictive models based on clinical and MRI radiomic features, as well as the methodology to produce these models. Methodology external validation showed maximal accuracy when models were retrained per-center using a locked methodology developed externally at another center. The achieved AUC_0.632+_ = 0.80 is comparable to previous results, with most studies reporting AUCs in the range of 0.73–0.87.^12,14–18^ Model external validation with feature harmonization and consolidation showed inferior accuracy with a maximum AUC = 0.70 achieved. Pooled data validation displayed a promising AUC_0.632+_ = 0.78, with balanced per-center performance.

### Model External Validation

The poor model external validation results before feature harmonization and consolidation indicate that critical differences exist between the 2 centers’ study samples. The clinical features predicting SRS response varied significantly between centers, with only GTV volume being a common predictor, as has been reported in other studies.^35,36^ All clinical features except sex were also significantly different between the 2 study samples, demonstrating that the samples are not comparable clinically. This is likely due to differences in year of treatment, clinical practice, and population demographics. These differences could also be caused by small sample sizes, as some less common primary cancer sites present at Center A were not present at Center B, whereas larger sample sizes could be more balanced. Further validation of clinical feature differences is required, especially considering the 5–17 year difference in SRS treatment dates between centers A and B. Other studies in the field have all been conducted using modern study samples that are therefore likely to be more similar to Center’s B dataset.^12–16,18–24^ Performing model external validation with Center B’s dataset and an external center dataset more representative of modern practice than Center A’s, could potentially show enhanced accuracy when models are transferred between centers.

### Methodology External Validation

The ability of Center A’s methodology to create an accurate model for Center B is an important step towards clinical translation, showing that a center could train a predictive model using a now-validated methodology. Given that clinical features across centers vary significantly and offered no benefit to Center B’s results, the use of only radiomic features is recommended for clinical implementation. This also eliminates the need for resource-intensive collection of clinical data. Furthermore, it is highly encouraging that methodology external validation was successful despite large inter-center differences.

### Pooled Data Validation

Pooled data model overall AUC values were always directly halfway between the AUCs for the models training for each center. The pooled model also provided AUCs within 0.04 when comparing accuracy between testing samples from Center A and Center B. This shows that multi-center models generalize across the centers on which they were trained, but a third center’s data is required to investigate the generalizability of these models.

### Feature Importance Analysis

Feature importance analysis revealed some features are predictive of SRS outcomes and resistant to inter-center variability. The number of highly important features also differed between the 2 centers (14 for Center A; 5 for Center B), which is likely due to Center A’s more diverse set of MR scanners compared to Center B.

Limited overlap in important radiomic features exists between our study’s centers and previous studies. Center A had a total of 4 highly important features overlap with other studies (kurtosis with 2 studies,^15,16^ and GLCM cluster shade and GLCM information measure correlation 2 both with one study^12^). Center B had 2 highly important features, range and neighboring gray-tone difference matrix strength, which overlap with another study.^16^ Across previous SRS studies, there are only examples of studies having a single important radiomic feature in common with another study.^12–16,18^ MR scanner, utilized MR sequences and regions-of-interest, clinical feature diversity, image preprocessing, and ML methodology variability likely all contribute to this finding, as our study controlled some of these variables and found greater radiomic feature overlap between centers.

### Feature Harmonization and Consolidation

ComBat harmonization did not improve model performance, and so it is not a viable solution for correcting inter-center variability, with other studies also demonstrating mixed effects.^37–39^ Before harmonization, nearly 80% of radiomic features were significantly different between centers, and so ComBat clearly brought these features into closer alignment, as demonstrated through more appropriate ROC operating point selection. ComBat’s utility despite performing MRI preprocessing shows that MRI differences were not fully corrected for by preprocessing. Our previous sensitivity study demonstrated that MR scanner variability within a single center negatively impacts model accuracy despite MRI preprocessing including Z-score intensity normalization.^17^ Most previous studies investigating predicting post-SRS/SRT progression using radiomics have a single MR scanner represented in their datasets,^12–14,18,23,24^ while the remaining studies have 2 scanners represented, with the effect of scanner variability not examined.^15,16,19,22^ MRI preprocessing and intensity normalization techniques vary widely across these previous studies as well, motivating the robust comparison of currently employed MRI normalization techniques and development of enhanced techniques to possibly improve model external validation accuracy.

The per-center feature importance analysis shows that ComBat failed because despite features being better aligned, the important radiomic features at each center were not necessarily important at both centers. Therefore, it was hypothesized that using a consolidated set of mutually important features was required, which did offer enhanced model external validation performance. Optimal performance occurring at *n* = 4 consolidated features likely indicates that using too few features does not provide enough degrees of freedom for accurate prediction, while too many features enables overfitting to a given center. This demonstrates a possible method for creating more generalizable models without retraining per-center, but external validation is needed, as the consolidated feature set was constructed using information from both centers.

### Limitations and Future Work

It is important to interpret the results of this study in the context of its limitations. First, both centers’ datasets were retrospective, leading to implicit inclusion and exclusion criteria being applied based on a center’s local population demographics and SRS treatment selection process. Since one follow-up MRI was required for study inclusion, patients who passed away shortly after SRS and those that declined or were not well enough for MRI were also systematically removed. Second, both centers’ datasets only included patients treated with linac-based SRS. No immediate conclusions can then be drawn about whether the methodologies, models, or results of this study would transfer to SRS-specific modalities (eg, Gamma Knife), and so future inclusion of datasets with patients treated with SRS-specific modalities is required. Third, a limited set of clinical features and MRI sequences was included to allow for both centers’ datasets to contain equivalent variables. Other clinical and MRI features have had predictive value in other studies,^14–16,18^ motivating further external validation with datasets of greater feature diversity. Fourth, interpretability of radiomic systems remains a barrier to clinical adoption. While our finding of consistently predictive features across 2 centers builds confidence in radiomic methods, discovering relationships between features and underlying biological mechanisms must be investigated.

The definition of our BM progression post-SRS endpoint is another limitation, as our method was nonstandard. This was required to include Center A’s dataset and also is a more robust volumetric method compared to the standard RANO-BM approach,^26^ but it does limit comparison to other studies. Our successful external validation reinforces our method’s validity, though future experiments should report both measures. The differentiation between true and pseudo-progression is also notoriously difficult.^40^ Our positive results suggest reasonable control of pseudo-progression, but prospective trials would allow for more reliable pseudo-progression determination, which may enhance model performance due to more accurate training datasets.

## Conclusions

We have demonstrated dual-center generalizability of models and modeling methodologies for predicting stereotactic radiosurgery outcomes using clinical and MRI radiomic features. We revealed that without radiomic feature harmonization and consolidation, a model trained at one center performs poorly at another. Next, we showed our model training methodology is generalizable by producing accurate models across both centers. We also showed that a pooled data model offers strong performance overall and balanced performance across the centers on which it was trained. The results of this external validation study provide crucial motivation for continued research to ultimately improve clinical outcomes in the treatment of brain metastases.

## Supplementary Material

vdad064_suppl_Supplementary_Data_S1

vdad064_suppl_Supplementary_Data_S2

vdad064_suppl_Supplementary_Material

## Data Availability

The ground truth label and predicted progression probability data from each reported experiment needed to replicate this study’s analysis are available at the following URL: https://github.com/baines-imaging-research-laboratory/radiomics-for-srs-external-validation-data-share. The computer code used to perform the reported machine learning experiments and subsequent analysis are available at the following URL: https://github.com/baines-imaging-research-laboratory/radiomics-for-srs-external-validation-code
