# Supplementary material for: Dual-center validation of using magnetic resonance imaging radiomics to predict stereotactic radiosurgery outcomes
Source: Neurooncol Adv. 2023 May 27;5(1):vdad064. doi: 10.1093/noajnl/vdad064 (PMC10289521; doi:10.1093/noajnl/vdad064)
Supplement: vdad064_suppl_Supplementary_Material [file vdad064_suppl_supplementary_material.pdf]

Table S1: Number of BMs imaged by each imaging configuration dependent on scanner model, acquisition orientation, and voxel size. The first two voxel size dimensions for each imaging configuration are the in-plane voxel dimensions, while the last voxel size dimension represents the slice thickness. Scanner manufacturers: Siemens (Erlangen, Germany), General Electric (Chicago, USA), Phillips (Amsterdam, The Netherlands)

Scanner Model and Field Strength	Acquisition Orientation	Voxel Size (mm^3)	# BMs (% +)
**Centre A**			
Siemens Magnetom Vision *(1.5 T)*	Sagittal	1×1×1.5	39 (28.2%)
Siemens Avanto *(1.5 T)*	Sagittal	0.5×0.5×1	37 (13.5%)
	Axial	0.5×0.5×2	8 (0.0%)
Siemens Magnetom Expert *(1.0 T)*	Sagittal	1×1×1.5	29 (31.0%)
Siemens Sonata *(1.5 T)*	Sagittal	1×1×1.5	5 (40.0%)
	Axial	1×1×1.5	1 (0.0%)
	Axial	1×1×2	3 (0.0%)
General Electric Signa HDxt *(1.5 T)*	Sagittal	1×1×1.5	1 (0.0%)
**Centre B**			
General Electric Optima MR450w *(1.5T)*	Axial	0.5×0.5×2	58 (12.1%)
	Axial	0.4×0.4×2	6 (16.7%)
General Electric Signa HDxt *(1.5 T)*	Axial	0.5×0.5×2	17 (23.5%)
	Axial	0.4×0.4×2	13 (23.1%)
	Axial	0.4×0.4×1	5 (0.0%)
Siemens Magnetom Aera *(1.5 T)*	Sagittal	1×1×1	8 (12.5%)
	Axial	0.7×0.7×5	3 (66.7%)
	Axial	0.4×0.4×0.9	2 (0.0%)
Siemens Avanto *(1.5 T)*	Axial	1×1×1	3 (66.7%)
	Axial	0.4×0.4×4	1 (0.0%)
Phillips Achieva *(1.5 T)*	Axial	0.4×0.4×3	1 (0.0%)

Table S2: Post-SRS progression, no progression and pseudo-progression calls for Centre B's dataset, broken down according to patterns of post-SRS BM size over time. For BMs with multi-step size over time patterns, sub-groups of BMs are described where clinical judgement was used in some cases to make a progression vs. pseudo-progression call. Progression scores marked with a "*" indicate BMs with complex clinical factors that make definitive progression calls difficult. These five BMs were possible confounders in model training, and so were removed for a specific test to gauge their effect. No effect was observed, and so the BMs remained in the dataset for the entire study.

Post-SRS BM size over time pattern	Clinical factors	# BMs	Progression score
1. Increase in size	Not applicable	5	Progression (+)
1. No increase in size	Not applicable	86	No progression (−)
1. Increase in size<br>2. Decrease in size	No other clinical factors	3	Pseudo-progression (−)
	Received immunotherapy or targeted agent shortly before decrease in size, but agents used were either deemed to have low brain penetration or unlikely to cause a decrease in size with as large of a magnitude as observed	3	Pseudo-progression (−)*
	Decrease in size caused by surgical resection, with surgical specimen pathology revealing radionecrosis and no cancerous progression	2	Pseudo-progression (−)
	Received salvage WBRT before the decrease was observed and so decrease was deemed to be caused by this additional radiation dose, indicating post-SRS cancerous progression had occurred	1	Progression (+)
1. Decrease in size<br>2. Increase in size	No other clinical factors	11	Progression (+)
	Immunotherapy was delivered before the observed increase, which was followed by a slight decrease (though it was not a 25% decrease, and so not recorded in the BM size pattern). The same behaviour was observed in the patient's second BM (see "Decrease, Increase, Decrease" pattern), and so it was deemed that cancerous progression had occurred post-SRS that was then controlled by the immunotherapy.	1	Progression (+)*
1. Decrease in size<br>2. Increase in size<br>3. Decrease in size	No other clinical factors	3	Pseudo-progression (−)
	Immunotherapy was delivered before the observed increase, which was followed by a decrease. The same behaviour was observed in the patient's first BM (see "Decrease, Increase" pattern), and so it was deemed that cancerous progression had occurred post-SRS that was then controlled by the immunotherapy.	1	Progression (+)*
1. Decrease in size<br>2. Increase in size<br>3. Decrease in size<br>4. Increase in size	The first increase was likely pseudo-progression, but the second increase was deemed indicative of cancerous progression after a complete response	1	Progression (+)

Table S3: Complete catalogue of the 107 radiomic features included within the study. All radiomic features were computed from the pre-treatment T1w-CE MRI, with complete documentation of the features provided by the PyRadiomics project (https://pyradiomics.readthedocs.io/en/latest/features.html). Abbreviations: GLCM (Gray-Level Co-occurrence Matrix), GLRLM (Gray-Level Run Length Matrix), GLDM (Gray-Level Dependence Matrix), GLSZM (Gray-Level Size Zone Matrix), NGTDM (Neighbouring Gray Tone Difference Matrix).

#	Feature Type	Feature Name
1	First-Order	10th Percentile
2	First-Order	90th Percentile
3	First-Order	Energy
4	First-Order	Entropy
5	First-Order	Interquartile Range
6	First-Order	Kurtosis
7	First-Order	Maximum
8	First-Order	Mean Absolute Deviation
9	First-Order	Mean
10	First-Order	Median
11	First-Order	Minimum
12	First-Order	Range
13	First-Order	Robust Mean Absolute Deviation
14	First-Order	Root Mean Squared
15	First-Order	Skewness
16	First-Order	Total Energy
17	First-Order	Uniformity
18	First-Order	Variance
19	Shape & Size	Elongation
20	Shape & Size	Flatness
21	Shape & Size	Least Axis Length
22	Shape & Size	Major Axis Length
23	Shape & Size	Maximum 2D Diameter Column
24	Shape & Size	Maximum 2D Diameter Row
25	Shape & Size	Maximum 2D Diameter Slice
26	Shape & Size	Maximum 3D Diameter
27	Shape & Size	Mesh Volume
28	Shape & Size	Minor Axis Length
29	Shape & Size	Sphericity
30	Shape & Size	Surface Area
31	Shape & Size	Surface Volume Ratio
32	Shape & Size	Voxel Volume
33	GLCM	Autocorrelation
34	GLCM	Cluster Prominence
35	GLCM	Cluster Shade
36	GLCM	Cluster Tendency
37	GLCM	Contrast
38	GLCM	Correlation
39	GLCM	Difference Average
40	GLCM	Difference Entropy
41	GLCM	Difference Variance
42	GLCM	Inverse Difference
43	GLCM	Inverse Difference Moment
44	GLCM	Inverse Difference Moment Normalized
45	GLCM	Inverse Difference Normalized
46	GLCM	Informational Measure of Correlation 1
47	GLCM	Informational Measure of Correlation 2
48	GLCM	Inverse Variance
49	GLCM	Joint Average
50	GLCM	Joint Energy
51	GLCM	Joint Entropy
52	GLCM	Maximal Correlation Coefficient
53	GLCM	Maximum Probability
54	GLCM	Sum Average
55	GLCM	Sum Entropy
56	GLCM	Sum Squares

#	Feature Type	Feature Name
57	GLRLM	Gray-Level Non-Uniformity
58	GLRLM	Gray-Level Non-Uniformity Normalized
59	GLRLM	Gray-Level Variance
60	GLRLM	High Gray-Level Run Emphasis
61	GLRLM	Long Run Emphasis
62	GLRLM	Long Run High Gray-Level Emphasis
63	GLRLM	Long Run Low Gray-Level Emphasis
64	GLRLM	Low Gray-Level Run Emphasis
65	GLRLM	Run Entropy
66	GLRLM	Run Length Non-Uniformity
67	GLRLM	Run Length Non-Uniformity Normalized
68	GLRLM	Run Percentage
69	GLRLM	Run Variance
70	GLRLM	Short Run Emphasis
71	GLRLM	Short Run High Gray-Level Emphasis
72	GLRLM	Short Run Low Gray-Level Emphasis
73	GLDM	Dependence Entropy
74	GLDM	Dependence Non-Uniformity
75	GLDM	Dependence Non-Uniformity Normalized
76	GLDM	Dependence Variance
77	GLDM	Gray-Level Non-Uniformity
78	GLDM	Gray-Level Variance
79	GLDM	High Gray-Level Emphasis
80	GLDM	Large Dependence Emphasis
81	GLDM	Large Dependence High Gray-Level Emphasis
82	GLDM	Large Dependence Low Gray-Level Emphasis
83	GLDM	Low Gray-Level Emphasis
84	GLDM	Small Dependence Emphasis
85	GLDM	Small Dependence High Gray-Level Emphasis
86	GLDM	Small Dependence Low Gray-Level Emphasis
87	GLSZM	Gray-Level Non-Uniformity
88	GLSZM	Gray-Level Non-Uniformity Normalized
89	GLSZM	Gray-Level Variance
90	GLSZM	High Gray-Level Zone Emphasis
91	GLSZM	Large Area Emphasis
92	GLSZM	Large Area High Gray-Level Emphasis
93	GLSZM	Large Area Low Gray-Level Emphasis
94	GLSZM	Low Gray-Level Zone Emphasis
95	GLSZM	Size Zone Non-Uniformity
96	GLSZM	Size Zone Non-Uniformity Normalized
97	GLSZM	Small Area Emphasis
98	GLSZM	Small Area High Gray-Level Emphasis
99	GLSZM	Small Area Low Gray-Level Emphasis
100	GLSZM	Zone Entropy
101	GLSZM	Zone Percentage
102	GLSZM	Zone Variance
103	NGTDM	Busyness
104	NGTDM	Coarseness
105	NGTDM	Complexity
106	NGTDM	Contrast
107	NGTDM	Strength

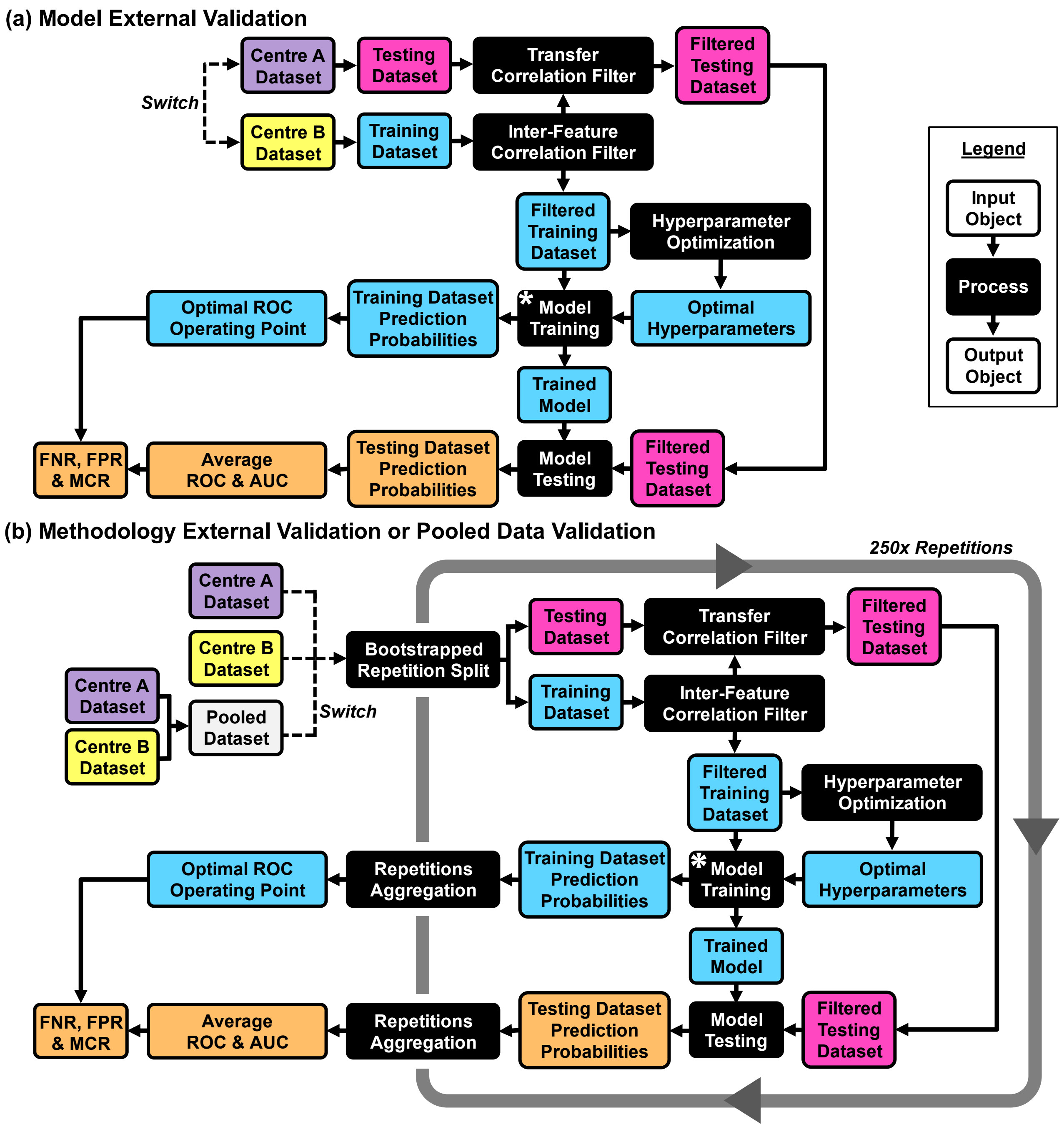

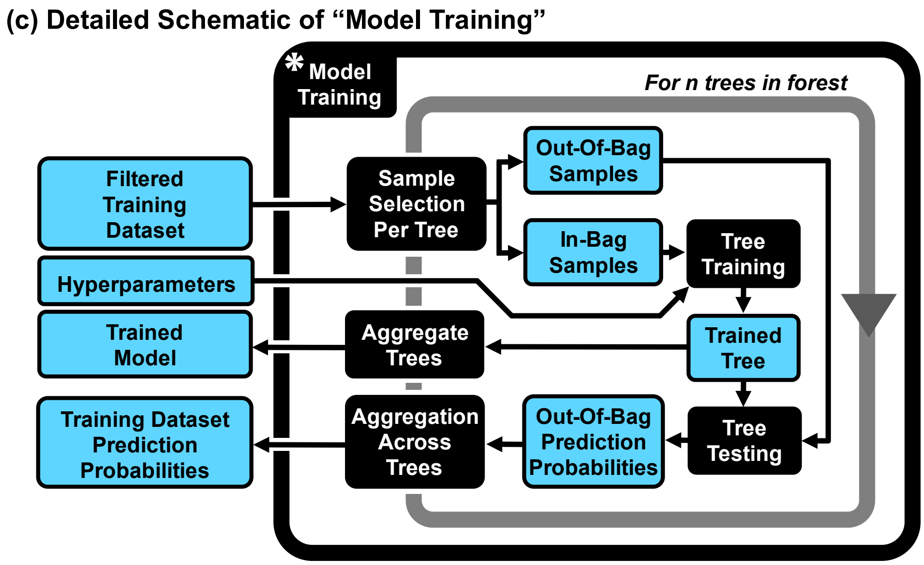

Figure S1: Schematic of the experimental design for (a) model external validation and (b) methodology external validation or pooled data validation. (a) can be performed with either Centre A or B's dataset as the training or testing dataset. (b) can be performed with Centre A's dataset, Centre B's dataset, or the pooled dataset. (c) shows a detailed view of the "Model Training" process present in (a) and (b). The schematics of (a) and (b) both demonstrate how training and testing datasets are kept separate until model testing to prevent data leakage.

Table S4: Hyperparameters for the random decision forest model used. For hyperparameters that underwent optimization, the optimization domain and search transform are provided. Numerical domains are indicated with minimum and maximum values in square brackets. Hyperparameter optimization was performed using 50 iterations of Bayesian optimization using the expected-improvement-plus acquisition function. The AUC on the out-of-bag samples was used as the optimization objective function. For hyperparameters that were not optimized, their value and justification are provided. For further descriptions of the hyperparameters, see the documentation for the *TreeBagger* function provided in Matlab 2019b.

Hyperparameter	Optimization Domain	Optimization Domain Search Transform
Number of trees	[10, 1000]	logarithmic
Number of features to sample	[1, number of features]	linear
Minimum leaf size	[1, number of features / 2]	logarithmic
Maximum number of decision splits	[1, number of samples – 1]	logarithmic
Feature selection	curvature, interaction curvature	categorical
Decision split criterion	Gini’s diversity index, deviance	categorical
	**Value**	**Justification**
In-bag fraction	1	produces in-bag dataset that is the same size as the training dataset
Sample with replacement	on	allows for out-of-bag samples to be reserved for evaluating trained model using the training dataset
Cost per SRS response	equal for each response	false negatives and positives given equal cost
Prior	empirical	allows priors to be optimized for the study population
Algorithm for categorical features	exact	all combinations of categories for categorical features investigated at decision splits
Merge leaves	off	leaf merging not needed as trees are not pruned
Prune	off	tree pruning not needed as the maximum number of decision splits hyperparameter was optimized
Surrogate decision splits	10	not all surrogate splits investigated to decrease model training time
Weights	equal for each sample	all samples given equal importance during training

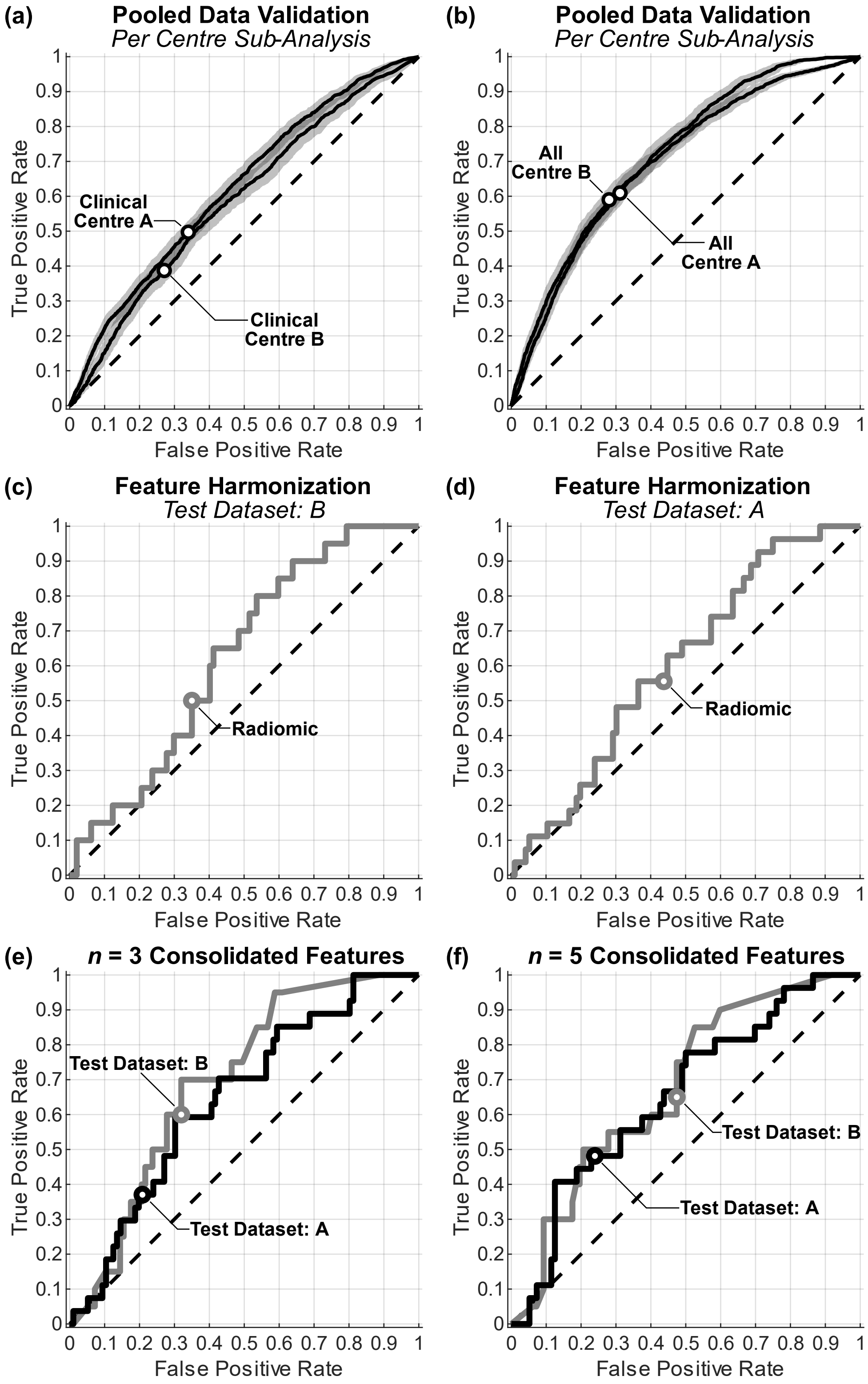

Figure S2: ROC curves and operating points for additional validation experiments. (a) and (b) show similar per centre sub-analysis as shown in Figure 2f, but for using only clinical features and using all features, respectively. (c) and (d) show the results of performing only ComBat harmonization for model external validation experiments using only radiomic features. (e) and (f) are identical to Figure 4b, but for the *n* = 3 and *n* = 5 cases of performing model external validation when feature harmonization and consolidation is applied.
